# Supplementary material for: Application value of contrast‐enhanced ultrasound in preoperative localization of microwave ablation for primary hyperparathyroidism
Source: J Appl Clin Med Phys. 2022 Oct 17;23(12):e13802. doi: 10.1002/acm2.13802 (PMC9797179; doi:10.1002/acm2.13802)

Application value of contrast-enhanced ultrasound in preoperative localization of microwave ablation for primary hyperparathyroidism

Fangyi Liu[1], MD, Li Zang[2], MD, Yunlin Li[1], MD, Zhiwei Guan[3], MD, Yang Liu[1], MD, Xiaoling Yu[1], MD, Zhiyu Han[1], MD, Ping Liang[1], MD

1. Department of Interventional Ultrasound, the First Medical Center, Chinese PLA General Hospital, NO.28 Fuxing Road, Beijing, 100853 China;
2. Department of Endocrinology, the First Medical Center, Chinese PLA General Hospital, NO. 28 Fuxing Road, Beijing, 100853 China;
3. Department of Nuclear Medicine, the First Medical Center, Chinese PLA General Hospital, NO. 28 Fuxing Road, Beijing, 100853 China;

Corresponding author: Ping Liang, MD, Department of Interventional Ultrasound, the First Medical Center, Chinese PLA General Hospital, NO.28 Fuxing Road, Beijing, 100853 China.

Tel.: +86 10 66939530.

Fax: +86 10 68161218.

E-mail address: liangping301@hotmail.com

E-mail address can be published.

Type of manuscript: Original research

Word count: 2600

Author Contribution Statement

Study conception or design: **Ping Liang, Fangyi Liu**

Analysis, or interpretation of data: **Yunlin Li, Yang Liu**

Drafting the work or revising: **Fangyi Liu**

Contributing patients and collecting data: **Li Zang, Zhiwei Guan, Xiaoling Yu, Zhiyu Han**

All authors read, revised, and approved the final manuscript.

Funding

This study has received funding by the National Scientific Foundation Committee of China (grant numbers 81871375).

Conflict of Interest

All authors declare that they have no conflicts of interest.

Data Availability Statement

The data that support the findings of this study are available on request from the corresponding author. The data are not publicly available due to privacy or ethical restrictions.

Acknowledgement

We thank all the participants in this study. We thank all the clinical staff who made sample collection possible.

Abstract

Background: Ultrasonography (US) and ^{99m}Technetium-sestamibi scintigraphy (^{99m}Tc-MIBI) are currently first-line imaging modalities to localize parathyroid adenomas with sensitivities of 80% and 84%, respectively. Therefore, finding other modalities to further improve the diagnostic accuracy for pre-operative localization is critically needed.

Purpose: To evaluate the application value of contrast-enhanced ultrasound (CEUS) in preoperative localization of microwave ablation for primary hyperparathyroidism (PHPT).

Methods: Between December 2012 and May 2021, 100 PHPT patients (34 males and 66 females; mean age, 56.31±13.43 years; age range, 25-85 years) with 130 suspected parathyroid nodules were enrolled. US, CEUS and ^{99m}Tc-MIBI were performed for the localization of pathological parathyroid glands. All patients were performed microwave ablation (MWA) under ultrasound guidance. All the suspected parathyroid nodules underwent core needle biopsy under ultrasound guidance during MWA to confirm the pathology. The diagnostic performance of all the imaging tests was analyzed in comparison with the pathological results.

Results: 130 nodules suspected to be of parathyroid origin from preoperative localization images were confirmed by pathological results, of which 116 were of parathyroid origin and 14 were not of parathyroid origin. The sensitivity, specificity, accuracy and AUC of CEUS in the localization of pathological parathyroid glands were 100%, 92.86%, 99.23% and 0.964, which were significantly higher than those of US (93.10%, 42.86%, 87.69% and 0.680) and ^{99m}Tc-MIBI (81.90%, 42.86%, 77.69% and 0.624) ($p<0.05$). The sensitivity and accuracy of CEUS were 100% and 97.22% which were higher than those of ^{99m}Tc-MIBI (65.62% and 63.89%) or US (75.00%

and 72.22%) in patients with multiple parathyroid glands ($p<0.05$). For smaller parathyroid adenomas (≤2 cm in diameter), the sensitivities of CEUS in locating hyperfunctioning parathyroid glands were 100%, which was significantly higher than that of ^{99m}Tc-MIBI (73.68% and 84.31%, $p<0.05$).

Conclusions: CEUS is a valuable preoperative localization method for PHPT patients performed MWA, especially for the patients with smaller pathological parathyroid gland and multiple glandular lesions.

Keywords:

Hyperparathyroidism, Ultrasonography, 99mTechnetium-sestamibi, Parathyroid neoplasms

Introduction

Primary hyperparathyroidism (PHPT) is known as a common endocrine disorder, and hypercalcemia is the main biochemical indicator of the disease [1,2]. Severe PHPT may lead to serious clinical symptoms such as pathological fracture, peptic ulcer, hypertension, myasthenia and other related diseases [3,4]. Because PHPT is mainly due to a solitary benign parathyroid adenoma, surgical resection is currently the main treatment in PHPT patients [4,5]. Minimally invasive treatment, including minimally invasive surgical resection and thermal ablation, has become possible and achieved a similar cure rate to traditional surgical resection [6-9]. Imaging of parathyroid glands plays an important role in localization of parathyroid adenomas prior to minimally invasive treatment[6,10]. Ultrasonography (US) and ^{99m}Technetium-sestamibi scintigraphy (^{99m}Tc-MIBI) are currently first-line imaging modalities to localize parathyroid adenomas with sensitivities of 80% and 84%, respectively [11,12]. However, these are related to the operator's experience and the patient's thyroid disease and surgical history[13-15]. Four-dimensional parathyroid computed tomography (4DCT) remains a problem-solving technique in challenging cases and after failed neck exploration, but it has radiation[16,17]. Therefore, finding other modalities to further improve the diagnostic accuracy for pre-operative localization is critically needed.

In recent years, contrast-enhanced ultrasound (CEUS) has aroused great interest as an imaging modality to improve the sensitivity and specificity of detecting and localizing abnormal parathyroid tissue [18,19]. In the setting of hyperparathyroidism, the glands are usually rich in blood supply. Typical peripheral blood supply or polar blood supply from the branch of the inferior thyroid artery can be seen [20]. CEUS can dynamically

detect microvascular perfusion, and microvascularization can be regarded as a reliable symptom to localize parathyroid gland adenomas in the preoperative period [21]. In addition, the research and development of perfusion analysis software can provide a quantitative information about tissue perfusion and then benefit the localization of hyperfunctional parathyroid glands [22]. Recent studies have shown CEUS can elevate the accuracy of preoperative localization of glands, especially in detecting double adenomas [18,21]. However, to our knowledge, the localization effect of CEUS in thermal ablation of PHPT is lacking. Therefore, the focus of this study was to clinically assess the value of CEUS in preoperative localization for PHPT patients performed microwave ablation(MWA) compared with conventional US and ^{99m}Tc-MIBI.

Materials and methods

Study design and patients

From December 2012 through May 2021, patients with hyperparathyroidism who underwent MWA under ultrasound guidance at our Department were enrolled. Exclusion criteria were secondary or tertiary hyperparathyroidism as well as allergy to contrast agent (Sonovue, Bracco, Italy). The diagnosis of PHPT was based on recommendations proposed by the International Workshop on Primary Hyperparathyroidism[3]. Parathyroid lesions were localized by US, CEUS and ^{99m}Tc-MIBI before ablation in all patients. Outcomes from the imaging modalities were reported as uniglandular disease, multiglandular disease, or negative. The ablated nodule was determined by the doctor who performed MWA based on the findings of all the localization imaging modalities. Ultrasound-guided core needle biopsy was performed for all the suspected parathyroid nodules on any of the localization imaging modalities during ablation to confirm the pathology. All imaging outcomes were

compared with pathological results. Cure was defined as a patient whose blood calcium level remained normal more than half a year after MWA.This study was approved by our institutional human research review committee. Written informed consent was obtained from all patients.

Preoperative localization imaging

All US examinations of the thyroid and the parathyroid gland region were accomplished by ultrasound experts with more than 15 years of experience in neck ultrasound using a multi-frequency linear probe. During the examination, we placed the patient in a supine position with their necks hyperextended, and tried to fully expose the front of the neck. B-scan US was used to examine the both sides of thyroid and parathyroid in the axial and longitudinal plane. Store the digital imaging for at least 30 s. Parathyroid lesion features, such as size, internal echo, margin and texture were evaluated. Color-coded Doppler sonography and power Doppler imaging were used for assessment of microvascularization.

CEUS was accomplished by an ultrasound expert with more than 15 years' experience in CEUS using a multi-frequency linear probe (6–9 MHz/LOGIQ E9/GE)， who are blinded to MIBI results. After the B-scan US and Doppler assessment of the suspected lesion, we selected the largest section of the lesion as the CEUS observation section and switched to CEUS mode. Dynamic CEUS was performed with a low mechanical index between 0.08 and 0.16. It is necessary to advise patients to avoid swallowing and speaking during imaging process. Each patient was given a rapid bolus injection of 2.4 ml sulphur hexafluoride microbubbles (Sonovue, Bracco, Italy) followed by 5 ml saline solution via a cubital venous cannula. The dynamic imaging sequences were documented from injection of contrast agent continuing to wash out. Imaging observation and acquisition time were no less than 3 minutes. Positive findings for

CEUS in suspected parathyroid adenomas were early arterial hypervascularization from the margin to the center plus washout in the late phase.

Dual-phase ^{99m}Tc-MIBI planar imaging was composed of early imaging and delayed imaging, which were obtained 30 min and 2 h after injection of 740 MBq ^{99m}Tc-MIBI intravenously, respectively. Planar images from the neck to the front chest were performed in a 128×128 matrix, 140 keV photo-peak, low-energy high-resolution parallel collimator. The typical finding of parathyroidism appeared as an area with increased ^{99m}Tc-MIBI uptake near the thyroid tissue in the early phase and become predominant with thyroid uptake and washout in the late phase. All imaging analyses were independently performed by two nuclear medicine specialists.

Statistical analysis

Data analysis was performed using SPSS17.0 for windows (SPSS Inc, Chicago, IL, USA) and the continuous data were expressed as mean ± standard deviation (SD) where data followed a normal distribution, or medians and interquartile range (IQR) where they did not. Based on the pathological results as the gold standard, the sensitivity (defined as the ratio of true-positive tests to the sum of true-positive and false-negative tests), specificity, accuracy, positive predictive value and negative predictive value of each of three localization imaging modalities were calculated. The McNemar test was used to compare the ratios of different imaging modalities. P values of <0.05 were regarded as statistically significant.

Results

Characteristics of population

A total of 110 patients met the inclusion criteria，while 10 were excluded because that were diagnosed as secondary hyperparathyroidism. Of the 100 patients with PHPT (34 males and 66 females; mean age, 56.31±13.43 years; age range, 25-85 years) who underwent MWA, all patients received preoperative localization using US, CEUS and ^{99m}Tc-MIBI (Figure 1). The flow chart of this study shown in Supplement Figure 1. In all 100 patients, a total of 130 nodules suspected of parathyroid origin in preoperative localization images were confirmed by pathological results, of which 116 were of parathyroid origin and 14 were of non parathyroid origin. 116 parathyroid gland lesions confirmed by pathology were found in 98 patients. No parathyroid gland lesions were found in two patients. One was thyroid adenoma and one was sympathetic ganglion (Horner syndrome after biopsy and MWA). In addition, the remaining 12 non parathyroid nodules were lymph nodes. The comparison between US, MIBI and CEUS regarding pathological results (PTH(pg/mL), Calcium(mmol/L), Phosphorus(mmol/L),ALP(IU/L) was shown in Supplement Table 1, 2, 3&4. Details regarding participation are presented in Table 1. The blood calcium of 96 patients remained normal for more than 6 months after MWA. The cure rate was 96%.

Localization results of US、CEUS and ^{99m}Tc-MIBI

CEUS correctly localized all abnormal parathyroid glands, while only 95 parathyroid lesions were detected by MIBI and 108 by US. The sensitivity, specificity, accuracy and Area under Receiver Operating Characteristic curve (AUC) of CEUS in the localization of pathological parathyroid glands were 100%, 92.86%, 99.23% and 0.964, which were significantly higher than those of US(93.10%, 42.86%, 87.69% and 0.680, $p<0.05$) and ^{99m}Tc-MIBI(81.90%, 42.86%, 77.69% and 0.624, $p<0.05$) (Table 2 and Figure 2). Using conventional US, pathological parathyroid glands in 8 cases were not correctly detected. In addition, among the parathyroid nodules

diagnosed by ultrasound, there were 6 lymph nodes, 1 thyroid nodule and 1 sympathetic ganglion. In addition, 21 pathologically confirmed abnormal parathyroid gland lesions were not detected by ^{99m}Tc-MIBI. The Characteristics of nodules with false-negative and false-positive diagnoses on ultrasound and MIBI were shown in supplement table 5. We found the median diameters of misdiagnosis nodules by US imaging and MIBI imaging were small (US 0.85cm & 0.95cm; MIBI 1.1cm & 1.1cm, respectively). And nodules are more likely to be misdiagnosed if they are in the lower left. Among the lesions diagnosed as parathyroid adenoma by MIBI and ultrasonography, there were 6 cases of misdiagnosis, respectively, which were verified to be lymph nodes (Figure 3). For the diagnosis of lymph nodes, CEUS has higher diagnostic sensitivity (91.7%), specificity (100%) and accuracy (99.2%) than conventional US (sensitivity, 50.0%, P=0.125; specificity, 93.1%, P=0.008; accuracy, 89.0%, P=0.001, respectively).

The diagnostic sensitivity of three imaging methods for different sizes of parathyroid nodules was analyzed. (Table 3). The sensitivity of CEUS was all 100% in three groups (d≤1; 1＜d≤2; d>2). For nodules larger than 2 cm in diameter, there was no difference in sensitivity among the three imaging methods. For parathyroid nodules less than 2 cm in diameter, especially parathyroid nodules less than 1 cm in diameter, the sensitivity of CEUS was significantly higher than that of ^{99m}Tc-MIBI ($P<0.05$), although there was no significant difference between US and CEUS.

The diagnostic ability of three imaging methods for patients with single and multiple gland diseases was analyzed (Table 4). Out of the 116 pathological parathyroid glands in 98 patients, 84 patients had unigland disease and 14 patients had multigland disease. The sensitivity and accuracy of CEUS were significantly higher than that of ^{99m}Tc-MIBI in patients with unigland disease or multigland disease($p<0.05$). Especially in

patients with multigland disease, the sensitivity and accuracy of CEUS were 100% and 97.22%, which were higher than those of US or ^{99m}Tc-MIBI ($p<0.05$).

Discussion

Parathyroid minimally invasive surgical resection or thermal ablation can effectively relieve the symptoms of bone and joint pain and muscle weakness and reduce the risk of fracture and cardiovascular calcification[8,9,23]. Therefore, the success rate of this operation and the recurrence rate after operation, to a certain degree, depend on an accurate preoperative localization of the abnormal parathyroid glands. Parathyroid adenomas usually get their blood supply from branches of the inferior thyroid artery[20]. CEUS has an advantage in dynamically detecting microcirculation. Therefore, CEUS has potential significance in the localization of parathyroid gland lesions, and it is of great significance to define its role in localization of pathological parathyroid glands. This study successfully demonstrated the feasibility of CEUS in the preoperative localization of MWA of PHPT for the first time.

In our study, the sensitivity, specificity, and accuracy of CEUS in the localization of pathological parathyroid glands were 100%, 92.86% and 99.23%, which were significantly higher than those of US (93.10%, 42.86% and 87.69%) and ^{99m}Tc-MIBI (81.90%, 42.86% and 77.69%) ($p<0.05$). ^{99m}Tc-MIBI and US are commonly used in clinical preoperative localization of abnormal parathyroid glands. Vijay et al. reported that US (80.80%, 92.35% and 75.73%) was superior to ^{99m}Tc-MIBI (71.82%, 94.61% and 69.00%) and SPECT (70.21%, 97.78% and 69.11%) in terms of sensitivity, positive predictive value and accuracy [24]. With its clinical application, CEUS gradually shows a unique diagnostic value compared with conventional US and ^{99m}Tc-MIBI. Uller W et al. compared the results of B-scan US and CEUS, and

showed that CEUS could accurately evaluate microcirculation of the parathyroid with both sensitivity and specificity of 98.4% [21]. Our study showed similar results to these previous studies. As to the hyperfunctioning parathyroid gland, we can see hypoechoic structure with uniform echogenicity and hypervascular on color doppler around the capsule and centrally in ultrasound and rapid contrast enhancement from the margin in the CEUS arterial phase compared to the thyroid [25]. In addition, CEUS can effectively distinguish between parathyroid glands and lymph nodes according to the enhancement characteristics. Parathyroid adenoma and Parathyroid hyperplasia presented with early and homogeneous hyperenhancement, with central wash-out in the later phases[26,27]. But Benign lymph nodes were observed centrifugal and homogenous enhancement, and a complete bright ring in the subcapsule of the lymph nodes[28,29].

The three imaging methods had different abilities in the localization diagnosis of parathyroid adenomas of different sizes. For nodules larger than 2 cm in diameter, there was no difference in sensitivity among the three imaging modes in our study. However, for parathyroid adenomas with a diameter of less than 2 cm, especially those with a diameter of less than 1cm, the sensitivity of CEUS was significantly higher than that of MIBI($P<0.05$), although there was no difference between US and CEUS, due to the limitation of sample size. Similar to what reports by other authors, Carral F et al reported size of removed adenoma ≤1 cm was an independent association between negative 99mTc-MIBI scanning[30]. ^{99m}Tc-MIBI results are also affected by patient's thyroid disease, body mass index(BMI) and surgical history [13-15,30]. Besides, gland weight could also affect the ^{99m}Tc-MIBI result, and the smaller the gland nodule, the more likely it is to be negative[31].

In our study, the sensitivity and accuracy of CEUS were significantly higher than that of ^{99m}Tc-MIBI in patients with single gland disease or multigland disease($p<0.05$). Especially in patients with multigland disease, the sensitivity and accuracy of CEUS are higher than US or ^{99m}Tc-MIBI ($p<0.05$). Bhansali A et al. in their reports described that the sensitivity and positive predictive value of single gland abnormality detected by US were 73% and 100%, respectively, while those of radionuclide imaging were 98% [12]. Among the patients with multigland disease, of the 10 abnormal parathyroid lesions detected by surgical exploration, 3 were missed by US and 6 were missed by radionuclide scan [12]. In our study, the sensitivity of US to detect a single adenoma was 98.1%, similar to other relevant experience using US [12,18]. Patients with multigland disease have a higher false negative rate of ^{99m}Tc-MIBI, which may be concerned with different functional activities of various abnormal glands, making the tracer less available for poorly functioning glands[12]. Compared with ^{99m}Tc-MIBI and US, CEUS is a better method of preoperative localization for patients with multigland PHPT. However, CEUS also has some limitations, especially in ectopic glands, so alternative imaging techniques will still be required. And sufficient medical equipment and skilled examiners are the prerequisite for accurate preoperative localization [32].

There are some limitations in our study. First, the number of non-parathyroid gland nodules was less. Studies with a larger sample size are needed. Second, the gold standard in this study is based on the pathological results of biopsy, not the pathology after bilateral exploration and resection of the parathyroid gland, and there is the possibility that location is missing on all imaging modalities, although this situation is rare.

Conclusion

CEUS is a valuable preoperative localization method for patients with PHPT, especially for those with smaller pathological parathyroid glands or/and multiple glandular lesions.

Abbreviations and acronyms:

contrast-enhanced ultrasound (CEUS), primary hyperparathyroidism (PHPT), microwave ablation (MWA), ^{99m}Technetium-sestamibi scintigraphy (^{99m}Tc-MIBI), Area under Receiver Operating Characteristic curve (AUC), Receiver Operating Characteristic curve(ROC)

Conflict of Interest:

All authors declare that they have no conflicts of interest.

Data availability statement:

The data that support the findings of this study are available on request from the corresponding author. The data are not publicly available due to privacy or ethical restrictions.

References

1. Suliburk JW, Perrier ND. Primary hyperparathyroidism. *The oncologist.* 2007;12(6):644-653.
2. Fraser WD. Hyperparathyroidism. *Lancet (London, England).* 2009;374(9684):145-158.
3. Bilezikian JP, Brandi ML, Eastell R, et al. Guidelines for the management of asymptomatic primary hyperparathyroidism: summary statement from the Fourth International Workshop. *The Journal of clinical endocrinology and metabolism.* 2014;99(10):3561-3569.
4. Bilezikian JP, Bandeira L, Khan A, Cusano NE. Hyperparathyroidism. *Lancet (London, England).* 2018;391(10116):168-178.

5. John P Bilezikian RM, Michael Levine, Claudio Marcocci, Shonni J Silverberg, John Potts. *The Parathyroids, 3rd Edition.* USA2014.
6. Insogna KL. Primary Hyperparathyroidism. *The New England journal of medicine.* 2018;379(11):1050-1059.
7. Udelsman R, Åkerström G, Biagini C, et al. The surgical management of asymptomatic primary hyperparathyroidism: proceedings of the Fourth International Workshop. *The Journal of clinical endocrinology and metabolism.* 2014;99(10):3595-3606.
8. Liu C, Wu B, Huang P, et al. US-Guided Percutaneous Microwave Ablation for Primary Hyperparathyroidism with Parathyroid Nodules: Feasibility and Safety Study. *Journal of vascular and interventional radiology : JVIR.* 2016;27(6):867-875.
9. Liu F, Yu X, Liu Z, et al. Comparison of ultrasound-guided percutaneous microwave ablation and parathyroidectomy for primary hyperparathyroidism. *International journal of hyperthermia : the official journal of European Society for Hyperthermic Oncology, North American Hyperthermia Group.* 2019;36(1):835-840.
10. Minisola S, Cipriani C, Diacinti D, et al. Imaging of the parathyroid glands in primary hyperparathyroidism. *European journal of endocrinology.* 2016;174(1):D1-8.
11. Kobylecka M, Płazińska MT, Chudziński W, et al. Comparison of scintigraphy and ultrasound imaging in patients with primary, secondary and tertiary hyperparathyroidism - own experience. *Journal of ultrasonography.* 2017;17(68):17-22.
12. Bhansali A, Masoodi SR, Bhadada S, Mittal BR, Behra A, Singh P. Ultrasonography in detection of single and multiple abnormal parathyroid glands in primary hyperparathyroidism: comparison with radionuclide scintigraphy and surgery. *Clinical endocrinology.* 2006;65(3):340-345.
13. Krausz Y, Lebensart PD, Klein M, et al. Preoperative localization of parathyroid adenoma in patients with concomitant thyroid nodular disease. *World journal of surgery.* 2000;24(12):1573-1578.
14. Barbaros U, Erbil Y, Salmashoğlu A, et al. The characteristics of concomitant thyroid nodules cause false-positive ultrasonography results in primary hyperparathyroidism. *American journal of otolaryngology.* 2009;30(4):239-243.
15. Haciyanli M, Lal G, Morita E, Duh QY, Kebebew E, Clark OH. Accuracy of preoperative localization studies and intraoperative parathyroid hormone assay in patients with primary hyperparathyroidism and double adenoma. *Journal of the American College of Surgeons.* 2003;197(5):739-746.
16. Zeina AR, Nakar H, Reindorp DN, et al. Four-dimensional Computed Tomography (4DCT) for Preoperative Localization of Parathyroid Adenomas. *The Israel Medical Association journal : IMAJ.* 2017;19(4):216-220.
17. Itani M, Middleton WD. Parathyroid Imaging. *Radiologic clinics of North America.* 2020;58(6):1071-1083.
18. Parra Ramírez P, Santiago Hernando A, Barquiel Alcalá B, Martín Rojas-Marcos P, Lisbona Catalán A, Álvarez Escolá C. Potential Utility of Contrast-Enhanced Ultrasound in the Preoperative Evaluation of Primary Hyperparathyroidism. *Journal of ultrasound in medicine : official journal of the American Institute of Ultrasound in Medicine.* 2019;38(10):2565-2571.

19. Agha A, Hornung M, Stroszczynski C, Schlitt HJ, Jung EM. Highly efficient localization of pathological glands in primary hyperparathyroidism using contrast-enhanced ultrasonography (CEUS) in comparison with conventional ultrasonography. *The Journal of clinical endocrinology and metabolism.* 2013;98(5):2019-2025.
20. Lane MJ, Desser TS, Weigel RJ, Jeffrey RB, Jr. Use of color and power Doppler sonography to identify feeding arteries associated with parathyroid adenomas. *AJR American journal of roentgenology.* 1998;171(3):819-823.
21. Uller W, Jung EM, Hornung M, et al. Evaluation of the microvascularization of pathologic parathyroid glands in patients with primary hyperparathyroidism using conventional ultrasound and contrast-enhanced ultrasound. *Clinical hemorheology and microcirculation.* 2011;48(1):95-103.
22. Platz Batista da Silva N, Jung EM, Jung F, Schlitt HJ, Hornung M. VueBox® perfusion analysis of contrast-enhanced ultrasound (CEUS) examinations in patients with primary hyperparathyroidism for preoperative detection of parathyroid gland adenoma. *Clinical hemorheology and microcirculation.* 2018;70(4):423-431.
23. Bleyer AJ, Burkart J, Piazza M, Russell G, Rohr M, Carr JJ. Changes in cardiovascular calcification after parathyroidectomy in patients with ESRD. *American journal of kidney diseases : the official journal of the National Kidney Foundation.* 2005;46(3):464-469.
24. Korwar V, Yuen Chang F, Teasdale E, Suchett-Kaye I, Edwards A, Morgan J. Stepwise Approach for Parathyroid Localisation in Primary Hyperparathyroidism. *World journal of surgery.* 2020;44(3):803-809.
25. Morris MA, Saboury B, Ahlman M, et al. Parathyroid Imaging: Past, Present, and Future. *Front Endocrinol (Lausanne).* 2021;12:760419.
26. Boudousq V, Guignard N, Gilly O, et al. Diagnostic performances of cervical ultrasound, sestamibi scintigraphy and contrast-enhanced (18)F-fluorocholine positron emission tomography in primary hyperparathyroidism. *Journal of nuclear medicine : official publication, Society of Nuclear Medicine.* 2021.
27. Pavlovics S, Radzina M, Niciporuka R, et al. Contrast-Enhanced Ultrasound Qualitative and Quantitative Characteristics of Parathyroid Gland Lesions. *Medicina (Kaunas).* 2021;58(1).
28. Chen L, Chen L, Liu J, Wang B, Zhang H. Value of Qualitative and Quantitative Contrast-Enhanced Ultrasound Analysis in Preoperative Diagnosis of Cervical Lymph Node Metastasis From Papillary Thyroid Carcinoma. *Journal of ultrasound in medicine : official journal of the American Institute of Ultrasound in Medicine.* 2020;39(1):73-81.
29. Wei Y, Yu MA, Niu Y, et al. Combination of Lymphatic and Intravenous Contrast-Enhanced Ultrasound for Evaluation of Cervical Lymph Node Metastasis from Papillary Thyroid Carcinoma: A Preliminary Study. *Ultrasound Med Biol.* 2021;47(2):252-260.
30. Berber E, Parikh RT, Ballem N, Garner CN, Milas M, Siperstein AE. Factors contributing to negative parathyroid localization: an analysis of 1000 patients. *Surgery.* 2008;144(1):74-79.
31. Garcia-Talavera P, Diaz-Soto G, Montes AA, et al. Contribution of early SPECT/CT to (99m)Tc-MIBI double phase scintigraphy in primary hyperparathyroidism: Diagnostic value and correlation between uptake and biological parameters. *Rev Esp Med Nucl Imagen Mol.* 2016;35(6):351-357.

32. Agha A, Hornung M, Rennert J, et al. Contrast-enhanced ultrasonography for localization of pathologic glands in patients with primary hyperparathyroidism. *Surgery*. 2012;151(4):580-586.

Figure legends

Figure 1. A 49-year-old female patient with HPHT. A. US shows a hypoechoic nodule behind the left thyroid lobe and CEUS shows high enhancement in the arterial phase (white arrow); B. 99mTc-MIBI scintigraphy shows a positive nodule in the left lobe of the thyroid (black arrow).

Figure 2 The ROC curves for MIBI, US and CEUS imaging modalities in the localization of parathyroid glands. The green curves represents the AUC of MIBI imaging and the AUC value is 0.624. The orange curves represents the AUC of US imaging and the AUC value is 0.680. The blue curves represents the AUC of CEUS imaging and the AUC value is 0.964.

Figure 3. A 51-year-old-female patient with HPHT. Pathology showed that the left lower pole lesion was lymph node and the right lower pole lesion was parathyroid adenoma. A. US shows a hypoechoic nodule behind each of the right thyroid lobe (i cross section, ii longitudinal section, white arrows) and the left thyroid lobe (iii cross section, iv longitudinal section, white arrows), considering parathyroid adenoma; B. The hypoechoic nodule at the right lower pole showed overall high enhancement in arterial phase (white arrows), considering parathyroid adenoma. C. CEUS showed that the lesions in the arterial phase of the left lower very hypoechoic nodule showed enhancement of hilus like structure (white arrows), considering lymph nodes;

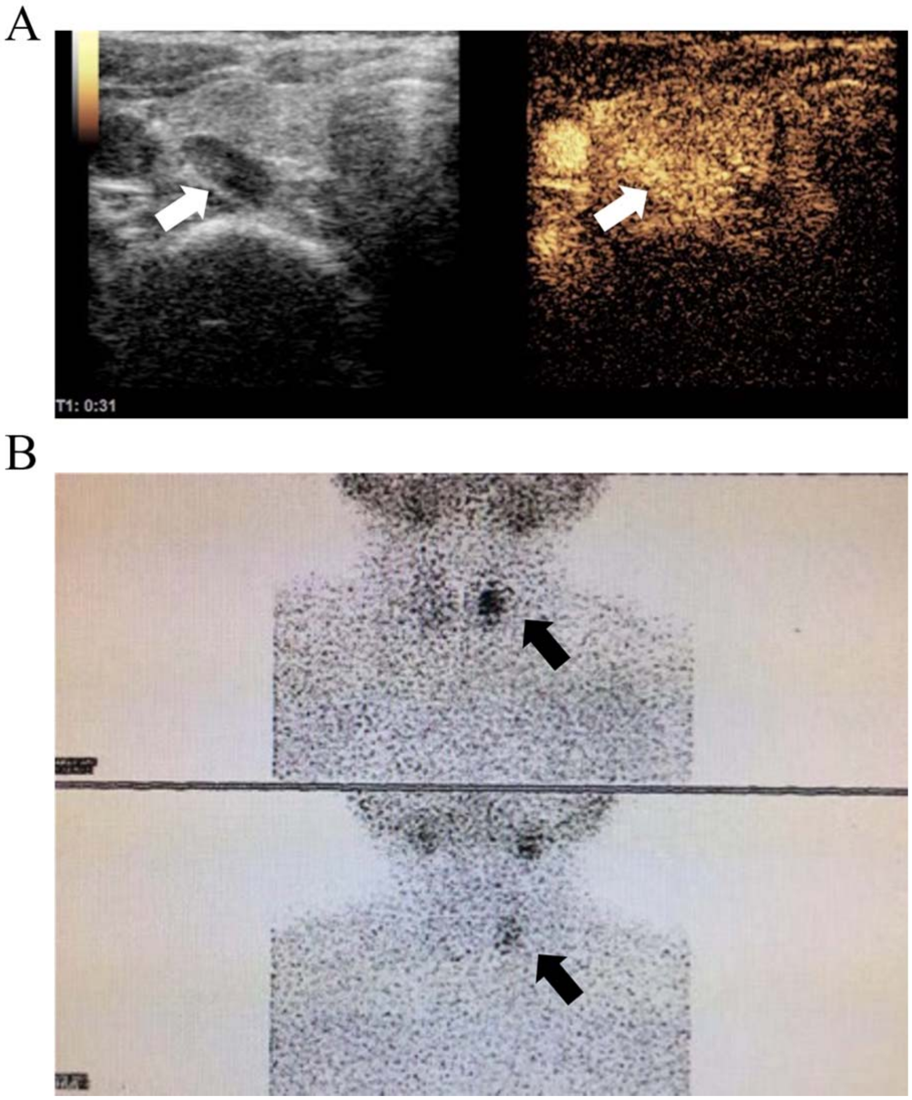

Figure 1. A 49-year-old female patient with HPHT. A. US shows a hypoechoic nodule behind the left thyroid lobe and CEUS shows high enhancement in the arterial phase (white arrow); B. 99mTc-MIBI scintigraphy shows a positive nodule in the left lobe of the thyroid (black arrow).

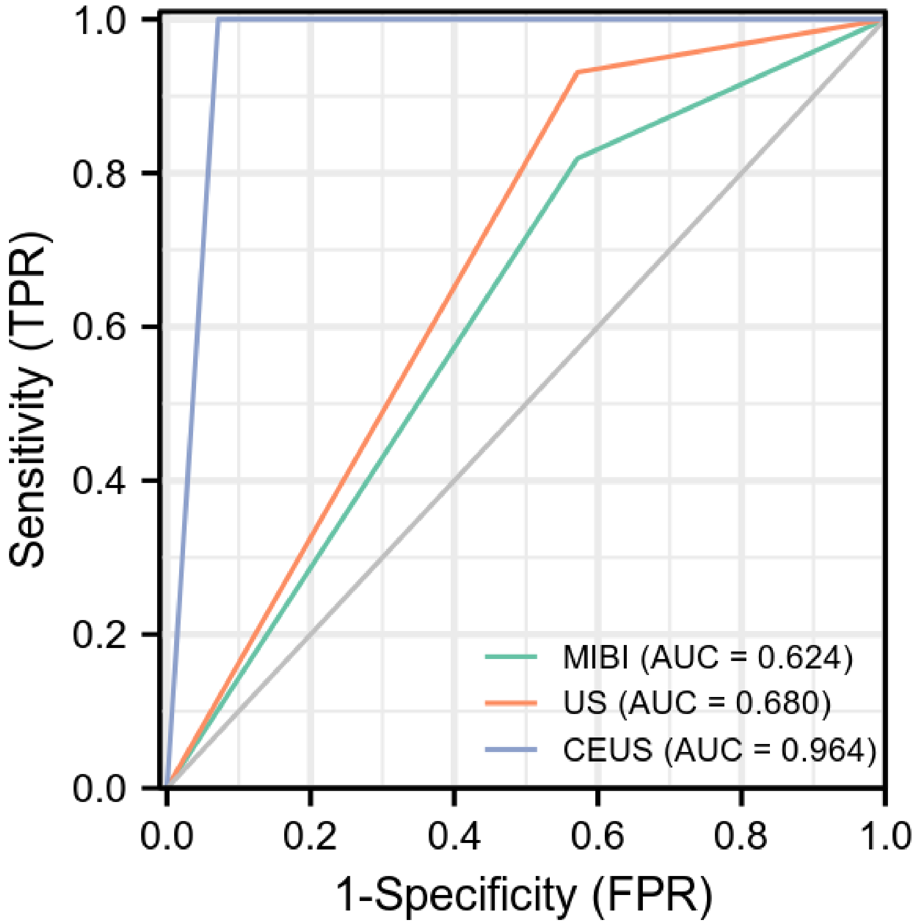

Figure 2 The ROC curves for MIBI, US and CEUS imaging modalities in the localization of parathyroid glands. The green curves represents the AUC of MIBI imaging and the AUC value is 0.624. The orange curves represents the AUC of US imaging and the AUC value is 0.680. The blue curves represents the AUC of CEUS imaging and the AUC value is 0.964.

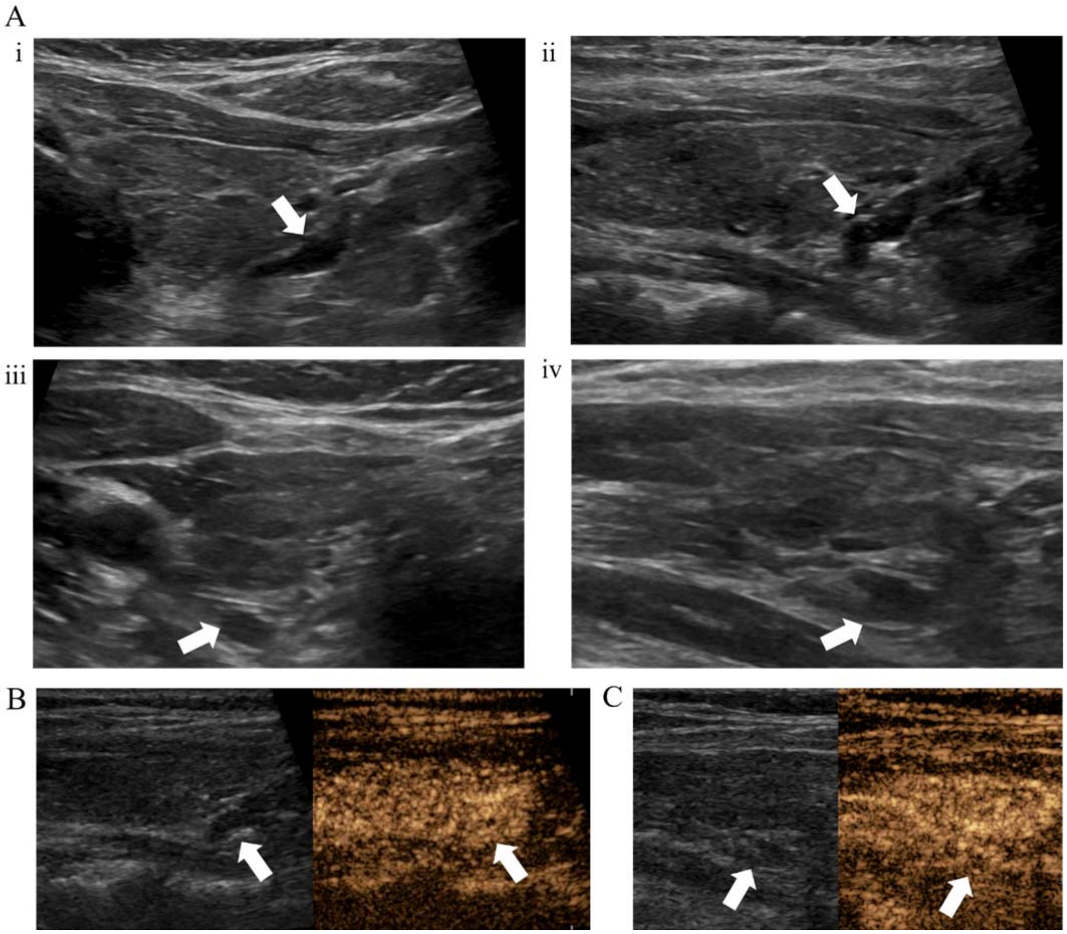

Figure 3. A 51-year-old-female patient with HPHT. Pathology showed that the left lower pole lesion was lymph node and the right lower pole lesion was parathyroid adenoma. A. US shows a hypoechoic nodule behind each of the right thyroid lobe (i cross section, ii longitudinal section, white arrows) and the left thyroid lobe (iii cross section, iv longitudinal section, white arrows), considering parathyroid adenoma; B. The hypoechoic nodule at the right lower pole showed overall high enhancement in arterial phase (white arrows), considering parathyroid adenoma. C. CEUS showed that the lesions in the arterial phase of the left lower very hypoechoic nodule showed enhancement of hilus like structure (white arrows), considering lymph nodes;

Table 1. Patient characteristics and preoperative laboratory findings.

Characteristics	Value
Patients (n)	100
Age(years)	56.31±13.43
Gender(male/femal)	34/66
Body mass index	23.57±3.03
Preoperative parathyroid hormone(pg/ml)	132.85(101.38,214.13)
Preoperative serum calcium(mmol/L)	2.64(2.48,2.84)
ALP(IU/L)	86.05(62.40,111.25)
Phosphorus(mmol/L)	0.89±0.22
25(OH)D3(ng/ml)	12.76±5.97
Patients with Unigaland/ Multigaland	84/14
Pathological findings	
Parathyroid nodules	116
Location	
Right	59
Left	57
Thyroid adenoma	1
Sympathetic ganglion	1
Lymph nodes	12

Note.—Data are means±SD or median with interquartile range (IQR) for continuous variables and are numbers of patients with percentages for categorical variables.Normal range: iPTH 15-65pg/ml, Calcium 2.09-2.54 mmol/L, Phosphorus: 0.89-1.6 mmol/L, ALP: 0-130 IU/L; 25(OH)D3 : 20-32ng/ml

Table 2. Comparison between CEUS, US and MIBI in all patients.

Parameters	US(%)	CEUS(%)	MIBI(%)	P value		
				US vs CEUS	US vs MIBI	CEUS vs MIBI
Sensitivity	93.10%	100%	81.90%	0.008	0.011	<0.001
Specificity	42.86%	92.86%	42.86%	0.039	1.000	0.039
Accuracy	87.69%	99.23%	77.69%	＜0.001	0.035	＜0.001
Positive predict value	93.10%	99.15%	92.23%			
Negative predict value	42.86%	100.00%	22.22%			

Table 3. Comparison between CEUS, US and MIBI sensitivity in patients with different lesion sizes.

Lesion diameter(cm)	US(%)	CEUS(%)	MIBI(%)	P value		
				US vs CEUS	US vs MIBI	CEUS vs MIBI
d≤1	86.84%	100%	73.68%	0.063	0.267	0.002
1<d≤2	96.07%	100%	84.31%	0.500	0.031	0.008
d>2	96.29%	100%	88.88%	1.000	0.625	0.250

Table 4. Comparison between CEUS, US and MIBI in patients with single and multiple gland disease.

	Parameters	US(%)	CEUS(%)	MIBI(%)	P value		
					US vs CEUS	US vs MIBI	CEUS vs MIBI
Unigaland	Sensitivity	100%	100%	88.09%	1.000	0.002	0.02
	Specificity	50.00%	100%	50.00%	0.125	1.000	0.125
	Accuracy	95.65%	100%	84.78%	0.125	0.031	＜0.001
	Positive predict value	95.45%	100%	94.87%			
	Negative predict value	91.30%	100%	28.57%			
Multigaland	Sensitivity	75.00%	100%	65.62%	0.008	0.581	0.001
	Specificity	50.00%	75.00%	50.00%	1.000	1.000	1.000
	Accuracy	72.22%	97.22%	63.89%	0.012	0.607	0.002

Positive predict value	92.31%	96.97%	91.30%
Negative predict value	20.00%	100%	15.38%

Supplementary Figure 1 The flow chart

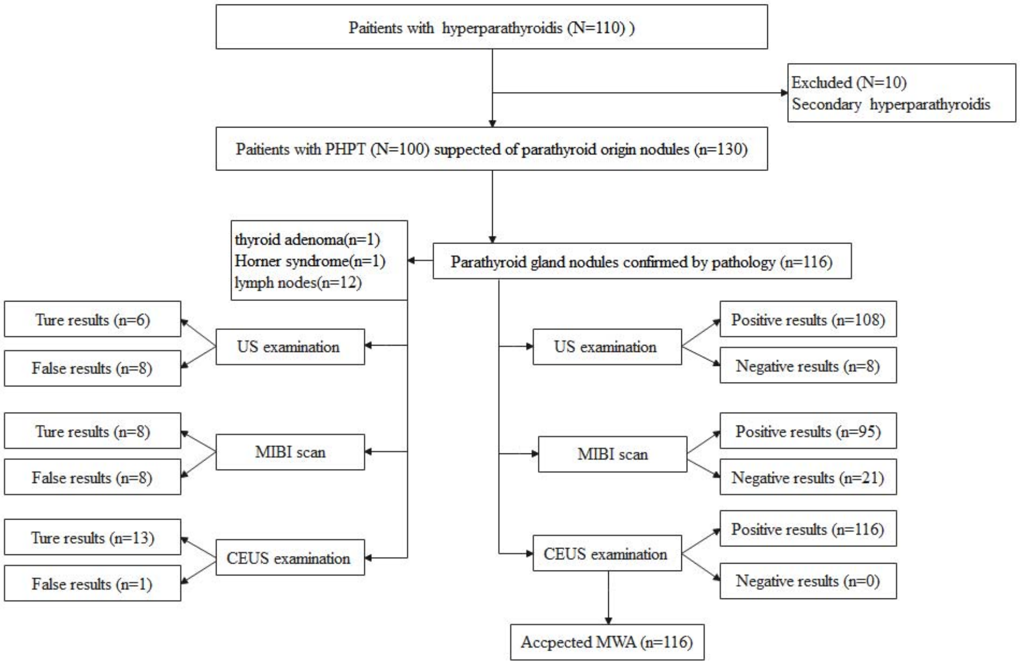

Supplement: Supplementary file 1 — Supporting Information [file ACM2-23-e13802-s002.pdf]
