# Supplementary material for: Application value of contrast‐enhanced ultrasound in preoperative localization of microwave ablation for primary hyperparathyroidism
Source: J Appl Clin Med Phys. 2022 Oct 17;23(12):e13802. doi: 10.1002/acm2.13802 (PMC9797179; doi:10.1002/acm2.13802)
Supplement: Supplementary file 2 — Supporting Information [file ACM2-23-e13802-s001.docx]

Supplementary Table 1 Dynamic change of PTH level in CEUS, US and MIBI group

| Time | CEUS | US | MIBI |
| --- | --- | --- | --- |
| 2h | 55.0317±62.3381 | 54.2023±62.2193 | 55.0317±62.3381 |
| 1 day | 47.6319±47.6319 | 45.3020±56.2792 | 47.6319±58.6173 |
| 1 month | 69.0757±34.8675 | 68.4142±34.1524 | 69.0757±34.8675 |
| 3 months | 62.3138±24.3202 | 61.8182±23.9264 | 62.3138±24.3202 |
| 6 months | 56.6398±23.9783 | 56.2943±23.5606 | 56.6398±23.9783 |
| 12 months | 51.5253±15.6278 | 50.7644±15.2910 | 51.5253±15.6278 |
| 24 months | 51.0112±21.8123 | 51.0112±21.8123 | 51.0112±21.8123 |

Supplementary Table 2 Dynamic change of Calcium level in CEUS, US and MIBI group

| Time | CEUS | US | MIBI |
| --- | --- | --- | --- |
| 2h | 2.5568 ± 0.2579 | 2.5635 ± 0.2541 | 2.5568 ± 0.2579 |
| 1 day | 2.4111 ± 0.2257 | 2.4086 ± 0.2231 | 2.4111 ± 0.2257 |
| 1 month | 2.3326 ± 0.1338 | 2.3272 ± 0.1314 | 2.3326 ± 0.1338 |
| 3 months | 2.3334 ± 0.1367 | 2.3293 ± 0.1394 | 2.3334 ± 0.1367 |
| 6 months | 2.3462 ± 0.1221 | 2.3428 ± 0.1232 | 2.3462 ± 0.1221 |
| 12 months | 2.3575 ± 0.0925 | 2.3519 ± 0.0919 | 2.3575 ± 0.0925 |
| 24 months | 2.3963 ± 0.1436 | 2.3963 ± 0.1436 | 2.3963 ± 0.1436 |

Supplementary Table 3 Dynamic change of Phosphorus level in CEUS, US and MIBI group

| Time | CEUS | US | MIBI |
| --- | --- | --- | --- |
| 2h | 0.9001 ± 0.2316 | 0.8979 ± 0.2291 | 0.900 ± 0.232 |
| 1 day | 1.0309 ± 0.2672 | 1.0329 ± 0.2652 | 1.031 ± 0.267 |
| 1 month | 1.0754 ± 0.1746 | 1.0764 ± 0.1761 | 1.075 ± 0.175 |
| 3 months | 1.0363 ± 0.1781 | 1.0493 ± 0.1857 | 1.036 ± 0.178 |
| 6 months | 1.0598 ± 0.1702 | 1.0654 ± 0.1706 | 1.060 ± 0.170 |
| 12 months | 1.0784 ± 0.1891 | 1.0875 ± 0.1886 | 1.078 ± 0.189 |
| 24 months | 0.9925 ± 0.1459 | 0.9925 ± 0.1459 | 0.993 ± 0.146 |

Supplementary Table 4 Dynamic change of ALP level in CEUS, US and MIBI group

| Time | CEUS | US | MIBI |
| --- | --- | --- | --- |
| 2h | 89.6735±46.6127 | 89.3587 ± 46.2037 | 89.6735±46.6127 |
| 1 day | 84.0958±37.8233 | 85.6514±37.7706 | 84.0958±37.8233 |
| 1 month | 94.5730±35.9342 | 94.1436±35.0787 | 94.5730±35.9342 |
| 3 months | 77.4367±30.2518 | 77.2175±29.7090 | 77.4367±30.2518 |
| 6 months | 75.5974±32.9275 | 75.4125±32.5237 | 75.5974±32.9275 |
| 12 months | 61.3800±13.0840 | 61.4364±12.4811 | 61.3800±13.0840 |
| 24 months | 80.3667±20.9037 | 80.3667±20.9037 | 80.3667±20.9037 |

Supplement Table 5 Characteristics of nodules with false-negative and false-positive diagnoses on ultrasound and MIBI

| Parameter | US | |  | MIBI | |
| --- | --- | --- | --- | --- | --- |
|  | False positive | False negative |  | False positive | False negative |
| Diameter,cm |  |  |  |  |  |
| Min | 0.6 | 0.4 |  | 0.7 | 0.6 |
| Median | 0.85 | 0.95 |  | 1.1 | 1.1 |
| Max | 2.3 | 2.3 |  | 2.5 | 3.2 |
| Mean | 1.0 | 1.1 |  | 1.5 | 1.3 |
| Nodule location |  |  |  |  |  |
| Upper right | 4 | 3 |  | 1 | 5 |
| Lower right | 0 | 0 |  | 1 | 4 |
| Upper left | 2 | 2 |  | 2 | 3 |
| Lower left | 2 | 3 |  | 4 | 9 |
